# Validation of Laser Capture Microdissection Protocol in Endometriosis Studies

**DOI:** 10.3390/medicina55090520

**Published:** 2019-08-22

**Authors:** Katiane de Almeida da Costa, Helena Malvezzi, Bruno Gallani Viana, Renée Zon Filippi, Rosa Maria Neme, Thiago Pinheiro Arrais Aloia, Sérgio Podgaec, Carla de Azevedo Piccinato

**Affiliations:** 1 Hospital Israelita Albert Einstein, São Paulo, BR 05652-900, Brazil; 2 Centro de Endometriose São Paulo, São Paulo, BR 04534-001, Brazil

**Keywords:** protein, RNA quality, microdissected area and histology

## Abstract

*Background and Objectives:* The presence of endometrial-like tissue outside the uterine cavity is a key feature of endometriosis. Although endometriotic lesions appear to be histologically quite similar to the eutopic endometrium, detailed studies comparing both tissues are required because their inner and surrounding cellular arrangement is distinct. Thus, comparison between tissues might require methods, such as laser capture microdissection (LCM), that allow for precise selection of an area and its specific cell populations. However, it is known that the efficient use of LCM depends on the type of studied tissue and on the choice of an adequate protocol. Recent studies have reported the use of LCM in endometriosis studies. The main objective of the present study is to establish a standardized protocol to obtain good-quality microdissected material from eutopic or ectopic endometrium. *Materials and Methods:* The main methodological steps involved in the processing of the lesion samples for LCM were standardized to yield material of good quality to be further used in molecular techniques. *Results:* We obtained satisfactory results regarding the yields and integrity of RNA and protein obtained from LCM-processed endometriosis tissues. *Conclusion:* LCM can provide more precise analysis of endometriosis biopsies, provided that key steps of the methodology are followed.

## 1. Introduction

Endometriosis is a disease characterized by the presence of endometrial-like tissue outside the uterine cavity [1]. Because of the morphological similarity between endometriotic lesions and normal endometrium, researchers have investigated both tissues to find characteristic genetic or protein markers related to the pathophysiology of endometriosis. However, the cellular composition of endometriosis and eutopic endometrium is distinct. Fewer glandular epithelial cells are found in endometriotic lesions than in eutopic endometrium or are even absent [2]. The stroma of endometriotic lesions is surrounded and infiltrated by immune, inflammatory and vascular system cells in numbers and types that are distinct from those found in the eutopic endometrium [3]. Considering that distinct cell types express various genes and proteins at different levels, comparisons between tissue samples with diverse cellular infiltrates would fail to accurately answer specific quests on gene and protein expression, especially when only a minimum amount of material is available for analysis.

The laser capture microdissection (LCM) is a technique used in molecular and cellular biology laboratories. By employing high quality resolution microscopy and laser cutting, LCM allows for the selection and excision of defined tissue areas and the study of specific cells populations. The precise selection of interest areas enables researchers to increase the sensitivity of subsequent molecular or protein analysis [4], thereby generating gene expression or protein profile data representative of in vivo physiological or pathophysiological environments [5]. Despite laser power and tissue processing adjustment (section thickness, staining, defrosting time, etc.), lasers might generate heat, which could accelerate DNA, RNA and protein degradation processes. Other factors such as UV light, chemicals, histological staining and enzymatic degradation (nucleases and proteases) may also contribute to cellular deterioration during LCM samples processing [6].

Tissue processing and LCM protocols depend on the tissue type, taking into consideration its texture and the presence of nucleases or proteases which, depending on the tissular concentration, may compromise RNA and protein integrity (e.g., pancreatic, spleen, lung) [4,7]. Therefore, the methodology needs adjustment depending on the tissue type in order to optimize the technique. Laser capture microdissection has been used in samples of various organs: pancreas [8], colorectal intestine [6], neural tissue [9], prostate [10] and also in endometrium [11,12] Recent studies have reported on the use of LCM and other microdissection technique variations in endometriosis [13,14,15] However, these studies have not examined the concentration, purity and integrity of RNA or protein obtainable after LCM tissue processing. In addition, as far as we know, there are no published reports on the possible influence of the different steps of LCM processing on the yield and quality of RNA or protein recovered from the tissue. Because RNA and protein extraction is considered protocol sensitive, it may be possible that any methodological modification could compromise the final quality of the analytes. We believe that optimizing the LCM processing steps can result in better molecular recovery and in an accurate analysis of selected tissue samples. Important advances to the understanding of the etiopathogenesis of endometriosis could be achieved, since it would be possible to study a specific area of interest or even isolate cells typically found in endometriotic lesions or in the endometrium.

The aim of the present work is to systematize the main steps involved in LCM processing of endometrial and endometriotic lesion biopsies in order to improve the amount and quality of the recovered material for RNA or protein analysis, as well as the viability of this material for further studies.

## 2. Materials and Methods

### 2.1. Participants—Eligibility Criteria

Ten and eight patients with endometriosis were, respectively, included for RNA and protein analysis. Eight patients without endometriosis as confirmed by videolaparoscopy were included. The inclusion criteria for all women were to be in their reproductive years, non-smoking and free from hormonal contraceptive medication for three months prior to inclusion in the study. Endometriosis was confirmed by histopathological examination of biopsies (collected during surgery) according to the criteria of the American Society for Reproductive Medicine [16]. Upon signing an Informed Consent Agreement approved by the Research Ethics Committee of the Hospital Israelita Albert Einstein (HIAE) (approved on 22 September 2017, number: 77409217.9.0000.0071), all patients provided eutopic endometrial biopsies, and/or endometriotic lesions. Biopsies were collected in surgery at the Surgical Center of HIAE.

### 2.2. Experimental Design

**Figure 1:**
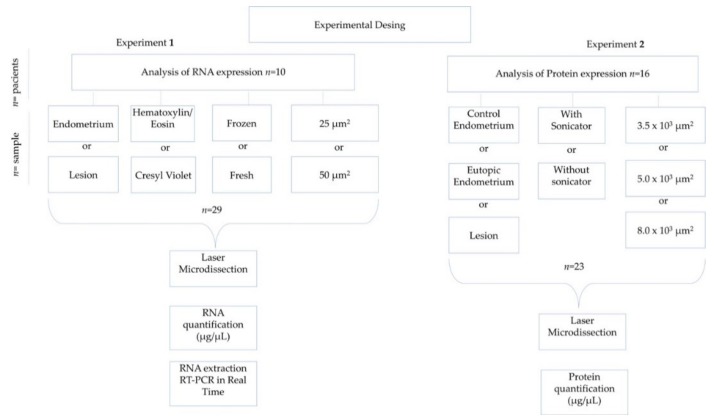
Schematic of the experimental design.

The tissue samples for RNA analysis were classified and compared according to: tissue type (endometrium or endometriotic lesion); staining protocol (Hematoxylin/Eosin (HE) or Cresyl Violet (CV)); fresh or frozen samples and respective preservation time after cryopreservation; and area (25 or 50 µm^2^). For protein concentration analysis, control endometrium (herein called eutopic endometrium) and endometriotic lesions from patients with endometriosis were analyzed, and the protein extraction methods (with or without sonicator) and areas (3.5 × 10^3^ μm^2^ or 5.0 × 10^3^ μm^2^ or 8.0 × 10^3^ μm^2^) were compared. A diagram of the experimental design is presented in Figure 1. During experimental analysis, the same researcher performed a single key procedure as, for instance, protein concentration measurements, RNA concentration and real-time PCR. However, for procedures such as microdissection, sample collection and protein extraction three different researchers performed the analysis to avoid the so called “experimenter bias”. Reproducibility was controlled using the same laser settings, the same tube–blade distance adjustment and the same microdissection time. Additionally, dye and alcohol used for tissue staining were made in large quantities at the beginning of the experiment, which was sufficient to be used for all experiments. In addition, prior to microdissection a morphological viability check was performed by pre-staining a single slide before mounting the microdissected specific slide. Intra-experimental variation was more clearly noticed when different types of microdissected tissue (endometrium or lesion) were compared.

### 2.3. Collection and Processing of Endometrial and Endometriotic Lesions Samples

The eutopic endometrial biopsies were collected at the beginning of the surgical procedure with Novak curette or with endometrial aspiration cannula, while a bipolar electrosurgical scalpel was used for lesion excision or biopsy during laparoscopic surgery. Different sorts of lesions were used. Most of them were deep-infiltrating lesions from the rectum and retrocervical ligament and endometriomas (ovarian lesions). Therefore, although different types of lesions were microdissected, all lesions were equally treated as lesions from where RNA and protein concentrations were measured, regardless of lesion type.

Eutopic endometrium and endometriotic lesion biopsies were immediately immersed in cryomedium agent Tissue-Tek OCT (Optimal Cutting Temperature) and the vials were placed in dry ice to be transported to the Research Laboratory of HIAE. Some fragments were kept at −80 °C for 3 days until processing (here called frozen samples), while other fragments of the same material were immediately processed (here called fresh samples).

### 2.4. Cryo-Sectioning

Samples embedded in OCT were serially sectioned into 10 μm sections on cryostat (Leica CM 1860, Wetzlar, Germany) at minus 25 °C. The cryosections were mounted on glass slides (MembraneSlide NF 1.0 PEN D Zeiss, Bernried, Germany), covered with a polyethylene naphthalate (PEN, Bernried, Germany) membrane (PEN1.0, Zeiss, Bernried, Germany) and were exposed to UV radiation for 20 min in order to help tissue fixation on the membrane.

### 2.5. Staining

Two staining protocols were tested: Cresyl Violet (CV) and Hematoxylin/Eosin (HE). Briefly, for CV staining, the sections were hydrated in decreasing alcohol solutions (100% and 70%) for 25 s in each solution. The CV was added to the slide for 30 s and finally the sections were dehydrated in increasing alcohol solutions (70% and 100%) for 25 s in each solution.

The HE staining protocol started with hydration of sections in decreasing ethanol solutions (100%, 90% and 70%) for 30 s each. The slides were washed in distilled water (DW) to remove excess alcohol, then immersed in hematoxylin for 30 s, washed in DW for 60 s, and placed in eosin for 3 s, washed with DW and finally dehydrated in increasing alcohol solutions (70%, 90% and 100%) for 30 s each.

### 2.6. Laser Microdissection

**Figure 2:**
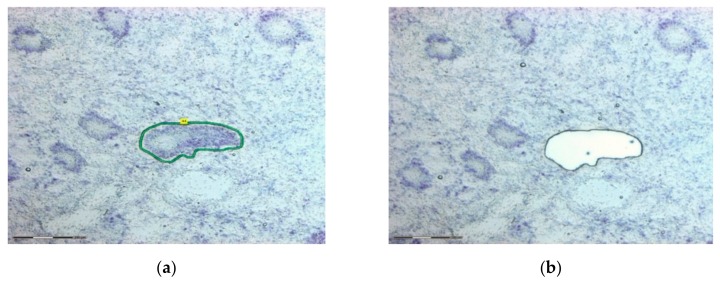
Histologic figure of representative samples before and after laser capture microdissection. This figure shows a specific area of the endometrium (**a**) being selected and (**b**) catapulted to the tube lid by the laser. Microdissected endometrial tissue at 5× magnification. Tissue stained with Cresyl Violet. Bar: 300 μm.

The laser capture microdissection was performed with computer system PALM RoboSoftWare 4.6 MicroBeam LSM 710, which consists of a source of laser beams adapted to an inverted optical microscope coupled to a video system. The cutting region, viewed in 20× objective, was carefully defined, considering a safety margin to ensure that the laser did not burn areas of interest with consequent loss of material (Figure 2). For this technique validation, general areas of endometrium and lesion were microdissected. The areas included epithelium, stroma, immune cells, vascular and other types of cells. Areas of 25 and 50 μm^2^ were microdissected for RNA analysis and areas of 3.5 × 10^3^ μm^2^, 5.0 × 10^3^ μm^2^ and 8.0 × 10^3^ μm^2^ were cut for protein concentration analysis. The microdissected material was catapulted into the adhesive covers of microdissection tubes (Sample AdhesiveCap 500 clear D Zeiss). Tube lids had either RNAlater or RIPA buffer containing 1% protease and phosphatase inhibitor (Thermo Fisher Scientific, Waltham, MA, USA), depending on the subsequent technique to be performed, RNA or protein extraction, respectively.

### 2.7. RNA Extraction

Total RNA was extracted from all microdissected samples using RNAaqueous Micro Total RNA Isolation Kit (Thermo Fischer Scientific, MA, USA), according to manufacturer’s instructions. In order to compare the amount of RNA recovered per sample, all extracted RNA samples were eluted in the same volume (15 µL) of water. The procedure was done in RNase free environment using RNAseZAP (Ambion, MA, USA), under ice. After extraction, the total RNA concentration was determined using a NanoDrop^®^ spectrophotometer (Thermo Fischer Scientific, MA, USA), at optical density 260 nm. Sample purity was checked by reading the plate between 230/260 nm and 260/280 nm. After quantification, total RNA was frozen at −80 °C until processing for transcription into cDNA.

### 2.8. Reverse Transcription

After treatment of the samples with DNase I (Sigma-Aldrich, St. Louis, MO, USA), complementary DNA (cDNA) was synthesized using a volume of 12 µL of total RNA per sample, irrespective of its concentration in the sample. All of the RNA obtained from each sample was used for the reverse transcription following the protocol of the Superscript III First-Strand Synthesis SuperMix kit (Invitrogen, Carlsbad, CA, USA). Water and RNA were briefly added in the final volume of 14 µL per sample. Samples were placed in thermocycler (Master Cycler-Nexus, Eppendorf, Hamburg, Germany) at 50 °C for 50 min to obtain cDNA. The reaction was stopped in ice and the cDNA stored at −80 °C.

### 2.9. Primers

A primer was designed to amplify GAPDH housekeeping gene sequence. GAPDH gene sequence was retrieved from the National Center database is Biotechnology Information (NCBI, Bethesda, MD, USA). The primer was designed from these sequences, using the Primer3 platform and tested with an in-silico PCR using the Genome Browser platform being: F: 5-GAAGGTGAAGGTCGGAGTCA and R-3: 5-3-TGAGGTCAATGAAGGGGTCA. GAPDH primer was synthesized with 100 bp by Invitrogen-Brazil. The primers’ efficiency was tested in triplicate by dilution curves of a cDNA pool obtained from endometriotic lesions and endometrium samples that were not microdissected.

### 2.10. qPCR (Polymerase Chain Reaction in Real Time)

SYBR Green qPCR assays were performed on 7500 Real Time PCR System (Applied Biosystems, Foster City, CA, USA) using SYBR^®^ Green Master Mix (Thermo Fisher Scientific, Waltham, MA, USA) detection system probes. A total of 8.4 μL of the mix solution, along with 0.84 μL of each primer (Forward and Reverse GAPDH), 3.42 μL of ultrapure water DNase/RNase free and 1.5 μL of cDNA were added, with a final volume of 15 μL per well of the plate. An extended number of cycle amplifications (50 cycles) were set in the PCR System to achieve amplification of even small amounts of RNA in the sample.

The specificity of the generated product was confirmed by analyzing the primers dissociation melting curve of the formed products. For gene expression adjustment between plates, a positive control was included in the experiment; in this case, a pool of non-microdissected endometriosis cDNA samples.

### 2.11. Electrophoresis

In order to check for PCR-real time amplification specificity as a way to complement the results from dissociation curves, a subset of samples (*n* = 11) was run in agarose gel electrophoresis. The agarose gel was run at 100 W voltage and stained with 2.5% ethidium bromide. The visualization was carried out using an ImageQuant 300 trans-illuminator (GE), coupled to a suitable photodocumentary system and software to capture 300 I Quant.

### 2.12. Protein Extraction

Total proteins were extracted from endometriosis and control microdissected samples. Fifty µL of RIPA buffer were added to each sample, vortexed for 1 min and centrifuged for 10 min at 4 °C at 10,000 rpm. The supernatant was harvested to another cryogenic tube. All samples contained the same final volume (50 µL). To compare protein extraction protocol, sonication was used in a subset of samples, always kept on ice to avoid any protein degradation. After extraction, the total protein concentration was calculated using Pierce’s method.

### 2.13. Protein Quantification

The supernatant’s protein lysates were quantified by Pierce’s method (BCA Protein Assay Kit, Thermo Fisher, Waltham, MA, USA). Briefly, in a 96 flat-bottom plate, 5 µL of the corresponding standard curve protein and of the samples were added in triplicate. Then, 100 µL of reagent B:A in 1:50 proportion were added to all wells. The plate was kept in a water bath at 37 °C for 30 min, and then read at Expectra Max adjusting absorbance to 562 nm after 2 s shaking.

### 2.14. Statistical Analysis

The data were analyzed using SPSS (IBM, Armonk, NY, USA) generalized estimation models, with log function, Gamma distribution and assuming correlation structure symmetry to contemplate the correlation between the different measurements of the same patient. The response variable was RNA concentration (ng/µL) and the dependent variables used to adjust the model were: tissue type (endometriotic lesions and endometrial), staining (CV and HE), type of preservation of samples (fresh or frozen) and microdissected area (25, 50 and 150 µm^2^). The data are presented as mean values accompanied by the 95% confidence interval and *p* value corrected by sequential Bonferroni method. For protein analysis GraphPad Prism 8.0 was used, as well as Mann–Whitney or Kruskal–Wallis multiple comparisons adjusted with Dunn’s test for non-parametric observations.

In all analysis, a *p* value of less than 0.05 was considered statistically significant.

## 3. Results

The RNA and protein concentration were analyzed to compare the yield obtained from endometriotic and eutopic tissue fragments varying in sampling amounts and submitted to various handling procedures. Moreover, amplification of GAPDH qPCR was used as a quality analysis tool of the RNA obtained after Laser Capture Microdissection.

### 3.1. Samples Purity Assessment

RNA sample purity was assessed by checking for DNA in agarose gels and by NanoDrop. No genomic DNA contamination was observed. NanoDrop sample measurements were between 2.0 and 2.2 arbitrary values, indicating samples free from reaction reagents.

### 3.2. RNA Concentration and Staining Protocols

**Figure 3:**
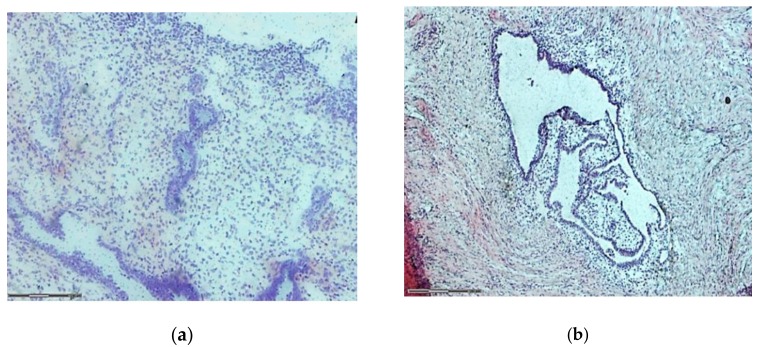
Microdissected endometrial tissue at 5× magnification. (**a**) Tissue stained with Cresyl Violet. (**b**) Tissue stained with Hematoxylin/Eosin. Bar: 300 μm.

**Figure 4:**
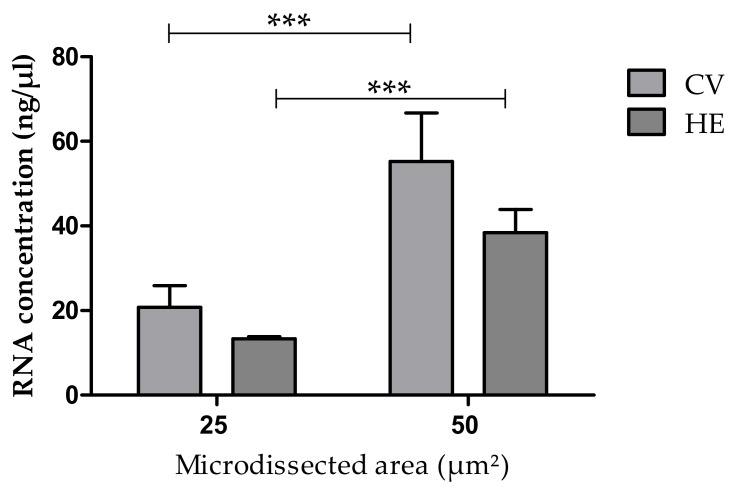
Concentration of RNA obtained from areas of 25 μm^2^ or 50 μm^2^ microdissected from endometrium sections stained with Cresyl Violet (CV) or with Hematoxylin/Eosin (HE). *** Bars indicate significant differences at *p* < 0.001.

We observed that both CV and HE can be used for staining slides in LCM protocols (Figure 3). No statistical difference was seen between microdissected endometrial and endometriotic lesions, regardless of preservation method (fresh or frozen) or the size of microdissected areas 25 μm^2^ (*p* = 0.152) and 50 μm^2^ (*p* = 0.185) (Figure 4). Time is a key factor to obtain better downstream results and faster processing steps are less harmful to samples [4]. Therefore, we chose the CV staining protocol for all subsequent analyses, since it demands fewer steps and thus, less time.

Significant difference (*p* < 0.001) was obtained for RNA concentration when comparing microdissected areas of 25 μm^2^ or 50 μm^2^ regardless of the previous staining technique (Figure 4) indicating that the RNA yield corresponds to the size of the dissected area.

### 3.3. RNA Concentration in Comparable Samples Obtained from Eutopic Endometrium or Endometriotic Lesions

**Figure 5:**
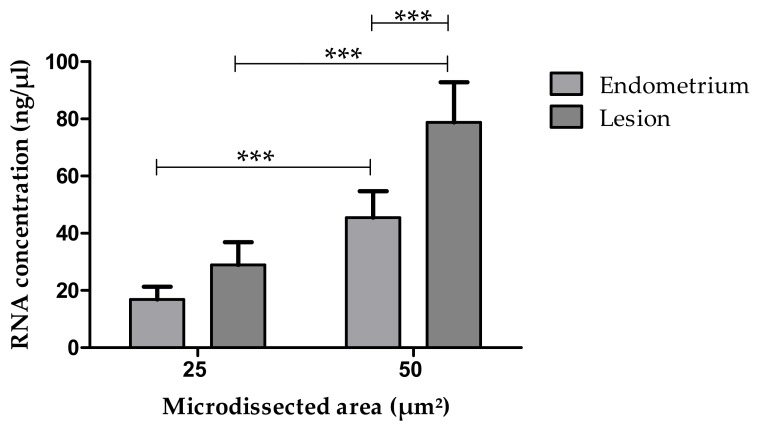
Concentration of RNA recovered from eutopic endometrium and endometriotic lesion relative to different microdissected areas (25 μm^2^ × 50 μm^2^). Different mRNA yield between the two tissue types was seen only for dissected areas of 50 μm^2^. Differences between microdissected areas of 25 μm^2^ versus 50 μm^2^ areas and respective RNA concentrations of either tissue are also shown. *** (*p* < 0.001).

When comparing RNA concentrations recovered from eutopic endometrium versus endometriotic lesion microdissected areas of 25 μm^2^, no statistical difference was seen *(p* = 0.094) (Figure 5). However, for an area of 50 μm^2^, a greater (*p* < 0.001) amount of RNA was recovered from endometriotic lesions as compared to the amount recovered from eutopic endometrial tissue (Figure 5).

Again, significant differences were observed when comparing microdissected areas of 25 μm^2^ and 50 μm^2^ (*p* < 0.001) of both tissues, although in this analysis previous preservation or staining methods were not taken into account (Figure 5).

### 3.4. RNA Content in Fresh or Frozen Tissue Samples

**Figure 6:**
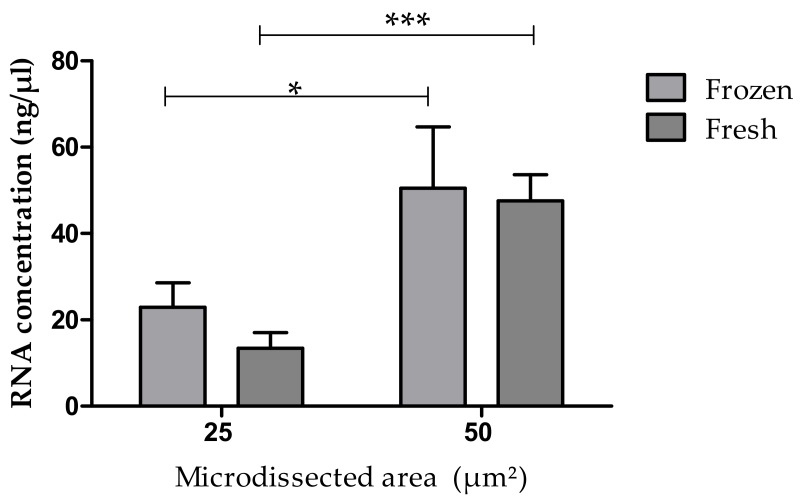
RNA concentration in fresh or frozen samples in microdissected areas of 25 μm^2^ and of 50 μm^2^. *** *p* < 0.001; * *p* = 0.004.

No statistical difference was seen in RNA concentrations obtained from analyzed areas of 25 μm^2^ and 50 μm^2^ in the comparison of frozen versus fresh samples (Figure 6). The dependence of the RNA yield on the excised area seen in the previous figures is maintained. This analysis did not take into account the previous staining method.

### 3.5. RNA Integrity

The means and standard deviation of CT (cycle threshold) for the housekeeping gene GAPDH were 35.2 ± 2.53 for microdissected areas of 25 μm^2^, and 31.94 ± 3.75 for the 50 μm^2^ areas. These values suggest that smaller microdissected areas require more cycles in order to be amplified.

### 3.6. Protein Concentration According to the Type of Analyzed Tissue

**Figure 7:**
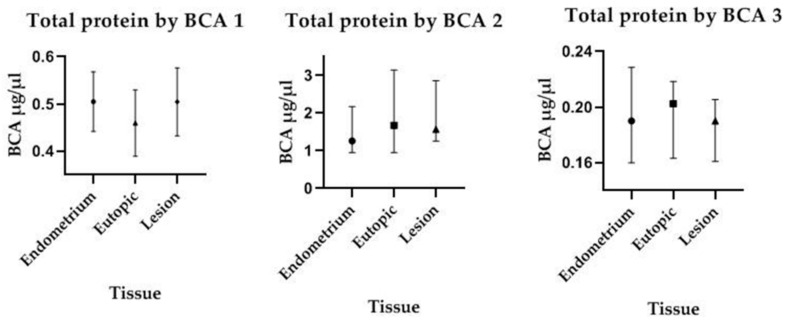
Protein concentrations measured by the BCA test at three different occasions. No statistically significant differences were observed among microdissected samples of identical size (5.0 × 10^3^ µm^2^).

Considering the same microdissected area (5.0 × 10^3^ µm^2^), no difference was found in total protein concentration among samples from endometrium (control group) and from eutopic endometrium or endometriotic lesions from patients. Although some variation occurred among experiments run at different occasions, protein recovery from the different tissues was similar within the same experiment, as shown in Figure 7.

### 3.7. Protein Concentration According to Microdissected Area

**Figure 8:**
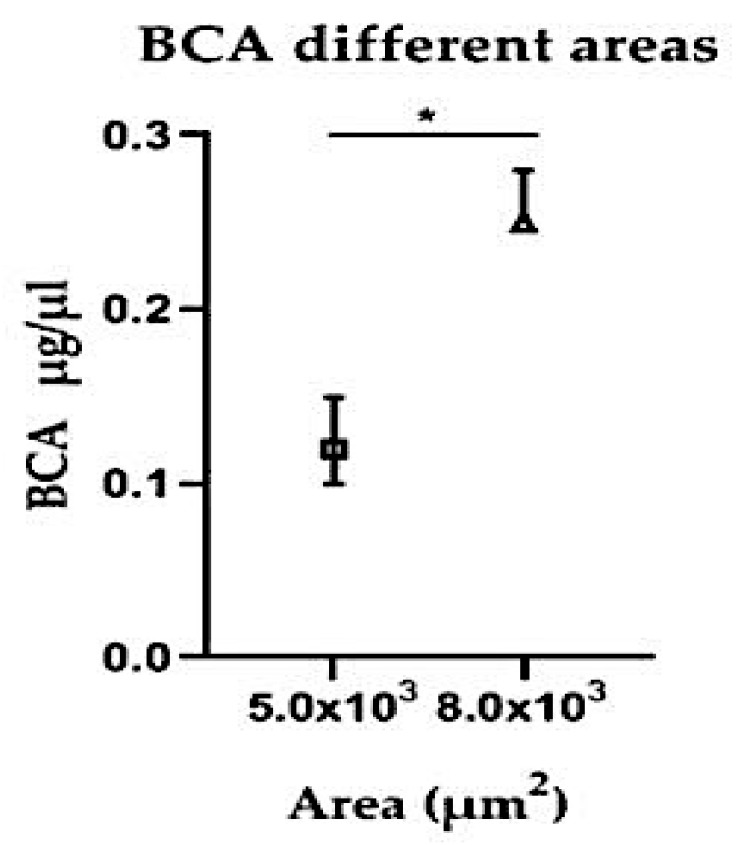
Protein concentration by BCA test in different size areas microdissected of the same tissue. * *p* = 0.0083.

To verify whether a moderate increase in the microdissected area would yield a verifiable increase in the protein concentration, and because larger protein amounts are often needed for quantifying scarce molecules, we increased the micro dissected area from 5 × 10^3^ µm^2^ to 8 × 10^3^ µm^2^ (circa 60%). It was observed that even this relatively small increase resulted in significantly more recovery of protein (Figure 8).

### 3.8. Protein Concentration and Sonication of the Microdissected Samples

**Figure 9:**
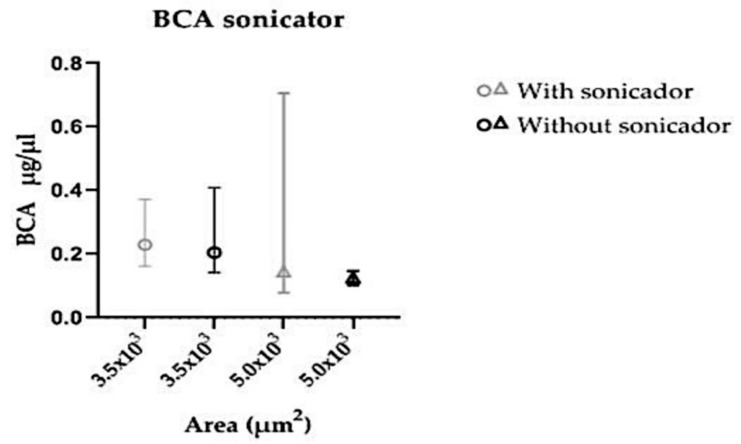
Comparison of protein concentration measured by BCA test in microdissected samples of endometrial tissue submitted to sonication or not prior to protein extraction.

To verify whether sonication would increase the protein yield, the samples were submitted to sonication prior to protein extraction. In the evaluated areas, 3.5 × 10^3^ μm^2^, and 5.0 × 10^3^ μm^2^, no statistical difference in protein concentration was seen among samples, whether previously sonicated or not (Figure 9).

## 4. Discussion

Cell-specific molecular investigations of endometriosis are essential to the understanding of its etiopathogenesis, but are hindered by technical difficulties. The LCM technique, by excising chosen tissue areas, allows for subsequent quantifying of DNA, RNA and protein of specific cell populations present in endometriotic lesions, as well as obtaining data on gene and protein expression. Even though endometriotic lesions and endometrial tissue are histologically similar, LCM potentially allows for the identification of molecular markers in specific cells, thereby providing clues on how endometriosis is regulated.

We have shown in the present work, for the first time, encouraging results on the recovery of RNA and protein from endometrium and endometriotic lesion tissue sections processed by LCM. The methodological protocols were adjusted to obtain material that would be suitable for further analysis. Several factors that could affect RNA and protein recovery and that might result in bias were controlled, especially considering that limitations in the amount or restriction of available material are common to researchers in endometriosis. Overall, we have demonstrated that the total RNA concentration (by NanoDrop) as well as the protein concentration (measured by BCA Protein Assay) obtained from microdissected sections with the use of LCM directly depend on the size of the excised area. The staining method (Cresyl Violet or HE), and whether the tissue sample was fresh or kept frozen before sectioning or the tissue type (endometrium or endometriotic lesion) did not influence the RNA and protein recovery. RNA quality was also assessed by amplification of GADPH, and checking for genomic DNA purity and the presence of contaminants and integrity (RT-PCR and agarose gel electrophoresis).

Tissue staining facilitates the identification of areas of interest and also the different types of cells and tissue structures. We corroborated the findings from Ordway et al. (2009) who also did not find differences in RNA integrity due to the staining used before LCM [9,12]. In contrast, Wang et al. (2006) found that the staining steps prior to LCM can affect the viability of the RNA [17]. Another LCM study on endometrial tissue compared two commercial staining kits. The CV-based kit was the one that gave the best results on final RNA integrity, which could be attributed in part to the fact that the used kit was alcohol-based, which may minimize the activity endogenous RNase [12]. Others [18] have found superior morphological quality in microdissected breast tissue of ruminant animals using the Arcturus HistoGene kit with the drawback of higher RNA degradation [18]. Conversely, Burgemeister et al. (2011) reported that staining with HE and CV ensures the best results in obtaining RNA from LCM in comparison to other staining methods [19]. Although no difference between the staining methods were seen in the present study, we chose to use the CV staining in all subsequent steps, because it is a faster protocol leading to good results in LCM [4].

Currently only one published study evaluated freezing time and the quality of RNA obtained by microdissection. It was, however, done on lactating ruminant’s breast tissue. The authors found that the tissue could be stored for several days at −80 °C without affecting the morphology and RNA quality, but freezing decreased the amount of material recovered [18]. The freezing method is effective at maintaining tissue viability, morphology and retaining the molecular composition of the sample. Indeed, frozen tissue is the best for obtaining high quality RNA [20]. But the RNases are reactivated when the tissue is thawed in an aqueous environment [8], and therefore, it is important to perform the entire procedure at low temperatures. Preservation of tissues at −80 °C is widely used, and able to preserve molecules such as proteins and DNA for many years. However, RNA preservation usually is not good after 5 years [21]. Thus, based on our results, we suggest that if it is not possible to perform LCM immediately after sample collection, the tissues should be frozen at −80 °C, and if possible, for no longer than 3 days, in order to ensure good recovery.

Research involving RNA or DNA quantitation and gene expression commonly does not require large amounts of material, since PCR enables amplification to such a degree that it allows for obtaining additional material for testing. However, currently, protein quantification methods are still limited, as they have low sensitivity to detect rare proteins. For this reason, larger sampling areas are needed in LCM for protein identification in downstream techniques [22].

The experiments carried out in the present study have shown that it is possible to obtain significant amounts of protein in microdissected endometrial tissue or endometriotic lesions. It is important to emphasize that the areas must be equal to, or greater than, 5.0 × 10^3^ μm^2^ and that there is no need to use additional processing for protein extraction (such as sonicating).

Nevertheless, the present study has some limitations. A single technique was used to measure protein concentration and no downstream experiments were executed in order to evaluate protein quality. However, we believe that the choice of a particular downstream technique for protein integrity assessment varies according to the original specific protein concentration. The choice of methods with different sensitivities, such as Western Blotting (less sensitive) or bead-based assays (as for example, Milliplex, more sensitive), depends on the concentration of the specific protein object of the study, by the mass of the hole tissue used for extraction [23]. Also, in the future, validation of the protocol used should be performed with a larger number of samples, considering types of lesion and types of microdissected cell.

## 5. Conclusions

We conclude that LCM can be used as a viable platform for the study of protein and gene expression in endometriosis. With the use of LCM in endometriosis, it will be possible to isolate areas of interest or separate different cell populations for molecular studies by adjusting the processing steps which precede the technique.
